# Prolactin and Autoimmunity

**DOI:** 10.3389/fimmu.2018.00073

**Published:** 2018-02-12

**Authors:** Vânia Vieira Borba, Gisele Zandman-Goddard, Yehuda Shoenfeld

**Affiliations:** ^1^Department “A” of Internal Medicine, Coimbra University Hospital Centre, Coimbra, Portugal; ^2^Faculty of Medicine, University of Coimbra, Coimbra, Portugal; ^3^Zabludowicz Center for Autoimmune Diseases, Sheba Medical Center, Tel-Hashomer, Israel; ^4^Sackler Faculty of Medicine, Tel-Aviv University, Tel-Aviv, Israel

**Keywords:** sex hormones, prolactin, autoimmunity, systemic lupus erythematosus, multiple sclerosis, systemic sclerosis

## Abstract

The great asymmetry of autoimmune diseases between genders represents one of the most enigmatic observations among the mosaic of autoimmunity. Sex hormones are believed to play a crucial role on this dimorphism. The higher prevalence of autoimmunity among women at childbearing ages, disease onset/relapses during pregnancy, and post-partum are some of the arguments that support this hypothesis. Certainly, motherhood represents one of the most remarkable challenges for the immune system, which not only has to allow for the conceptus, but also has to deal with complex endocrine alterations. Hormonal homeostasis is known to exert a crucial influence in achieving a competent and healthy immune system. Prolactin (PRL) has a bioactive function acting as a hormone and a cytokine. It interferes with immune system modulation, mainly inhibiting the negative selection of autoreactive B lymphocytes. Likewise, hyperprolactinemia has been described in relation to the pathogenesis and activity of several autoimmune disorders. Dopamine is an effective inhibitor of PRL secretion due to either a direct influence on the hypophysis or stimulation of postsynaptic dopamine receptors in the hypothalamus, arousing the release of the PRL inhibitory factor. Hence, dopamine agonists have proven to offer clinical benefits among autoimmune patients and represent a promising therapy to be explored. In this review, we attempt to provide a critical overview of the link between PRL, autoimmune diseases, and motherhood.

## Introduction

Currently, more than 80 autoimmune disorders are recognized, in which aberrant immune responses against self-different organs and tissues play a crucial role (1). Gender dimorphism represents one of the most enigmatic observations among the mosaic of autoimmunity. Susceptibility genes, epigenetic modifications, gender-related composition of gut microbiota, and sex hormones are believed to be a mainstay of this asymmetry (2, 3). The greater prevalence of autoimmunity among childbearing age women, disease relapses during pregnancy, and post-partum are some of the arguments that support this hypothesis (4). Indeed, women have enhanced immune reactivity, larger antigen-presenting capability and mitogenic responses, increased antibody production, higher immunoglobulin (Ig) levels, and the ability to reject allografts more rapidly (5). The immune and neuroendocrine system are intimately connected, partaking of dynamic bidirectional communication. Prolactin (PRL) has a recognized immune-stimulatory effect, specially inhibiting the negative selection of autoreactive B lymphocytes, promoting autoimmunity. In accordance, hyperprolactinemia has been associated with several autoimmune diseases, influencing its pathogenesis (6). Although the mechanisms involving this interaction are not completely understood, it has been documented that PRL can influence the communication and regulation of immune cells (7).

## PRL, the Hormone, and the Cytokine

Prolactin is a 23-kD peptide hormone secreted in the pituitary gland, through the hypothalamic–pituitary–adrenal axis, under tonic inhibition of dopamine. Interestingly, this hormone can also be produced in extra-pituitary locations, such as decidua, ovary, prostate, mammary gland, adipose tissue, brain, and immune cells. When produced in extra-pituitary sites, PRL has different molecular weight and bioactivity. Hyperprolactinemia is usually defined as fasting levels of above 20 ng/ml in men and above 25 ng/ml in women (8). The expected rate among healthy population is up to 3%. Levels physiologically increase during lactation, but also as result of several diseases, including prolactinoma, hypothyroidism, and adrenal insufficiency (9). Besides, PRL secretion is regulated by cytokines such as interleukin (IL)-1, IL-2, and IL-6, which are stimulators, while endothelin-3 and interferon (IFN)-γ play an inhibitory role. This hormone can be found adopting several isoforms due to variations in posttranslational modifications (10). The three main isoforms are the monomeric (free little PRL), big PRL, and macroprolactin (big big). The most biologically potent isoform is the monomeric free (little) PRL, which consists of 199 amino acids and has a molecular weight of 23 kDa (11). The PRL receptor is a member of the type 1 cytokine/hematopoietic receptor superfamily and is widely expressed through the immune system, including monocytes, lymphocytes, macrophages, natural killer cells, granulocytes, and thymic epithelial cells (12). Hence, the binding of PRL to its receptor activates downstream signaling pathways that will manipulate immune cells proliferation, differentiation, secretion, and survival (13, 14). This molecule is an integral member of the immune-neuroendocrinology network and has been largely associated with autoimmune diseases (15).

### PRL and Immune Modulation

Prolactin strongly persuades the innate and adaptive immune responses, managing the maturation of CD4− CD8− thymocytes to CD4+ CD8+ T cells, through IL-2 receptor expression (16, 17). A direct correlation between PRL levels and the number of B and CD4+ T lymphocytes has been reported (18). Indeed, hyperprolactinemia can impair B-cell clonal deletion, deregulate receptor editing and diminish the threshold for activation of B cells, promoting auto-reactivity (19–21). It is capable of changing Th1 and Th2 type cytokine production, promoting IL-6 and INF-γ secretion, and playing a regulatory role on IL-2 levels (22, 23). Furthermore, PRL increases Ig production, stimulates the development of antigen-presenting cells expressing major histocompatibility complex class II, and upholds the co-stimulatory molecules CD86, CD80, and CD40 (24). Interestingly, assorted autoantibodies, including anti-cardiolipin, anti-PRL, anti-La, anti-Ro, among others, were detected in patients with hyperprolactinemia (25–27). Finally, PRL has been shown to influence dendritic cells to skew antigen presentation to pro-inflammatory function phenotype, enhancing IFN-α production (28). During pregnancy, one of the most decisive immunologic adaptations is the shift from a Th1/Th17 pro-inflammatory response toward a Th2/T regulatory cell (Treg) response, which promotes tolerance and inhibits natural killer cells cytotoxicity (2, 29). In accordance, differences in the activity of assorted autoimmune diseases have been reported during pregnancy and post-partum. For a better comprehension, the effects of PRL on the immune system cells were summarized in Table 1.

**Table 1:** Effects of prolactin (PRL) on the immune system cells.

Immune cells	PRL secretion	Prolactin receptor (PRLR) expression	Immunological effects of PRL	Reference
Thymocytes	✓	✓	Promote the differentiation of CD4− CD8− thymocytes into CD4+ CD8+ cells Regulate the maintenance of thymocyte viability during differentiation	(30, 31)
Dendritic cells	?	✓	Enhance the production of cytokines (IL-12, TNF-α, IL-1β) Increase the responsiveness in allogeneic mixed leukocyte reactions (upregulation of MHC surface expression and the co-stimulatory molecule CD80)	(32, 33)
T cells	✓	✓	Exert an immunomodulatory role at early stages of T-cell activation Increase secretion of TNF-α, IFN-γ, and IL-2 Trigger the IL-2-stimulated proliferation Promote dysfunction of regulatory T cells Enhance adhesion to endothelial cells	(34–37)
B cells	✓	✓	Influence B-cell maturation process, promoting the survival of self-reactive clones Increase the viability of immature B cells by rescuing them from apoptosis	(38, 39)
Natural killer cells	?	✓	Induce natural killer cells differentiation to PRL-activated killer cells (PAK cells) in a dose-dependent way Interfere with proliferation and cytotoxic activity Promote the release of IFN-γ	(40–42)
Monocytes	✓	✓	Increase TNF expression	(43–45)
Granulocytes	?	✓	Activate the STAT1 and MAPK pathways Contribute for the transcription of IRF-1 and iNOS	(46, 47)
Macrophages	✓	✓	Cooperate with other pro-inflammatory stimuli to activate macrophages *via* engagement with the PRLR Promote the secretion of chemokines and cytokines (IL-1β, IL-12β, IFN-γ, and TNF)	(7, 48–50)

*iNOS: inducible nitric oxide synthase; IFN, interferon; IL, interleukin; IRF-1, interferon regulatory factor 1; MAPK, mitogen-activated protein kinase; MHC, major histocompatibility complex; STAT1, signal transducer and activator of transcription 1; TNF, tumor necrosis factor*.

### PRL during Pregnancy and Breastfeeding

Sex hormones can influence different functions on the immune system network. Typically, PRL and estrogens act as immune stimulants, while progesterone and testosterone exert a suppressive role (51, 52). Pregnancy inspires unique changes in endocrine and immune signaling, in order to tolerate and support the development and survival of the placenta and fetus in the hostile maternal immune system environment. PRL levels increase during pregnancy and reach peak values during delivery (53). Suckling stimulates the nerve endings in the nipple-areolar complex and strongly promotes hormone production. A large study performed by Stuebe et al. (54) evaluated PRL levels in women who exclusively breastfed their infants. The authors successfully reported wide changing baseline values (from 9 ng/dl before to 74 ng/dl 10 min after breastfeeding), depending on the frequency of feedings (54). In accordance, during the pregnancy and lactation period, several patients experience disease onset or relapse, suggesting an active influence of PRL. Indeed, a significant association between PRL levels and disease activity was found in systemic lupus erythematosus (55), rheumatoid arthritis (50, 56), and peripartum cardiomyopathy patients (57, 58), therefore breastfeeding should not be encouraged among those patients.

### PRL and the Role of Dopamine Agonists

Dopamine is an effective inhibitor of PRL secretion due either a direct influence on the hypophysis or stimulation of postsynaptic dopamine receptors in the hypothalamus, arousing the release of the PRL inhibitory factor. Bromocriptine is an ergot alkaloid that binds to the dopamine receptor and inhibits the central synthesis of PRL. In addition, this drug can also influence T and B lymphocytes through the dopamine receptor (59, 60). Bromocriptine has been shown to decrease autoantibodies production, influence lymphocyte function and modulate the expression of surface molecules. By contrast, it exerts no clear effect on extra-pituitary PRL production. In conclusion, the beneficial therapeutic effects in murine and human trials, and the low toxicity of the drug outline a solid rationale for its attempt in future therapeutic proposals (61).

## Hyperprolactinemia and Autoimmune Diseases

Hyperprolactinemia has been reported in patients with several autoimmune diseases, commonly manipulating disease development and perpetuation (62). The link between PRL and autoimmunity has been proposed to have a genetic background (63, 64). The PRL gene is located on the short arm of chromosome 6, near the HLA-DRB1 region, which is known for its association with assorted immune-mediated disorders (65).

### PRL and Systemic Lupus Erythematosus

Systemic lupus erythematosus is an autoimmune disease, typically affecting young women at reproductive age (66). Hyperprolactinemia has been reported in a wide range of lupus patients from both genders (15–33%). In accordance, PRL levels have shown direct correlation with clinical and serological disease activity (16, 67–69). Results from several trials report also an association with neurological, renal and hematological involvement, serositis, enhanced anti-double-stranded DNA antibodies, and diminished complement (70, 71). Furthermore, PRL bolsters the development of lupus-like phenotype in non-prone mice and exacerbated the disease in a lupus murine experimental study (72). During pregnancy, hyperprolactinemia has been associated with lupus anticoagulant, disease activity, and poor outcomes for mother and fetus (73). In accordance, the presence of anti-PRL antibodies was correlated with lower disease activity and better outcomes in pregnant patients (74, 75). The treatment of pregnant women with bromocriptine was shown to prevent disease relapses, improve outcomes, and reduce the doses of concomitant steroidal therapy (76, 77). In conclusion, the evidence strongly supports the role of PRL in the pathogenesis and activity of systemic lupus erythematosus.

### PRL and Anti-Phospholipid Syndrome

Anti-phospholipid syndrome is a systemic autoimmune condition, characterized by thrombotic events and/or pregnancy morbidity in the presence of anti-phospholipid antibodies. Hyperprolactinemia was detected in 12% of patients with anti-phospholipid syndrome, with no differences among genders or disease subtypes. Likewise, hormone levels were shown to be correlated with the presence of lupus anticoagulants, intrauterine growth retardation, and miscarriages among pregnant patients (78). By contrast, no significant correlation was found with thrombotic events, although PRL was recently proposed as a novel risk factor for thrombotic disease, since it acts as a potent platelet aggregation co-activator (79–82). Previously, bromocriptine was tested in animal models with anti-phospholipid syndrome and lupus, showing a suppressive effect on both diseases, probably through induction of natural non-specific CD8 suppressor cells (59).

### PRL and Rheumatoid Arthritis

Rheumatoid arthritis is a chronic autoimmune disease that if untreated leads to progressive and irreversible destruction of cartilage and bone. The relationship between PRL and rheumatoid arthritis emerged from the adjacent location of the human PRL gene and HLA region (16). Recent studies reported higher levels of PRL in serum and synovial fluid of patients with rheumatoid arthritis. This suggests increased production, either systemic or locally secreted by immune cells, in putative relation with disease activity (45, 56). Pregnant women with rheumatoid arthritis, due to a transient period of hypercortisolism, experience disease improvement. After delivery, flares are frequently reported (83). Women who breastfeed after the first pregnancy have a higher risk of developing rheumatoid arthritis, suggesting an active influence from PRL (84, 85). In addition, nearly 90% of these women will relapse within the first 3 months of postpartum and almost all patients will flair within the next 9 months. Indeed, severe disease was associated with longer breastfeeding periods and higher number of breast fed children. In animal models, bromocriptine was able to suppress postpartum exacerbation of collagen-induced arthritis (86). In humans, the treatment with bromocriptine revealed controversial findings (87, 88), probably because bromocriptine does not influence lymphocyte-derived PRL production (89). Hence, systemic and locally produced PRL may offer distinct contributions to inflammatory arthritis.

### PRL and Systemic Sclerosis

Systemic sclerosis is a connective tissue disease characterized by alterations of the microvasculature, disturbances of the immune system, and massive deposition of collagen and other matrix substances in the skin and internal organs (90). High levels of PRL have been reported in 13–59% of patients with systemic sclerosis (91). Likewise, a significant correlation between hormone levels and the severity of skin sclerosis, lung, and cardiovascular involvement was found (92–94). The sources of PRL in this disease are believed to reside on enhanced lymphocytic secretion, increased dopaminergic central tone, and drug-induction, mainly by antidepressants and prokinetics (95). Pregnancy *per se* does not exacerbate the disease, even though cases have been reported of women with organ insufficiency mainly pulmonary hypertension and severe skin fibrosis (96, 97). Patients with disease duration of less than 4 years, with diffuse cutaneous subtype, presence of anti-RNA polymerase III or anti-topoisomerase I antibodies are at higher risk for obstetric complications and should delay pregnancy until the disease is quiescent (98). In conclusion, PRL was found to be correlated with disease severity and activity.

### PRL and Multiple Sclerosis

Multiple sclerosis is a chronic inflammatory disorder involving the central nervous system (99, 100). In animal models, it is represented by experimental autoimmune encephalomyelitis, believed to be an inflammatory response against oligodendrocytes that form myelin sheaths surrounding neuronal axon driven by myelin-reactive CD4+ Th1/Th17 cells (101). Several studies reported a positive correlation between hyperprolactinemia and disease onset, relapse, and number of anti-myelin oligodendrocyte glycoprotein antibody secreting cells (102, 103). Indeed, the source of high PRL levels among those patients is unclear, albeit observations suggest that it may be part of a non-specific hypothalamic*–*pituitary–adrenal axis dysregulation due to neurodegeneration and/or demyelination (104). Currently, PRL is believed to have a dual impact in the central nervous system. On the one hand, PRL might support system repair by providing regenerative signals for neurons, oligodendrocytes, and adult neural stem/progenitor cells. On the other hand, its stimulation of peripheral immune cells might promote aberrant immune responses and negatively impact the disease (105, 106). Typically, pregnancies were believed to have a negative impact in women with multiple sclerosis, provoking postpartum exacerbations and increasing permanent disability (107). Nowadays, it is known that the risk of relapse significantly declines during the third trimester of pregnancy and increases three-fold in the first 3–4 months after delivery, with no references about medication consumption or breastfeeding options (108). Recently, studies revealed that an earlier return of menses was associated with a higher risk of disease relapse in the first 6 months after delivery, which suggests a natural protection from exclusive breastfeeding (109). Likewise, prolonged lactational amenorrhea was correlated with a lower risk of postpartum relapses (110). In conclusion, evidence supports a plausible protection from exclusive breastfeeding, although no studies have examined the long-term effects of breastfeeding, particularly in exclusive patterns.

#### PRL and Celiac Disease

Celiac disease is a gluten-sensitive autoimmune enteropathy where both adaptive immunity and innate immunity are involved in its development (111). Serum PRL levels were positively correlated with disease activity, degree of mucosal atrophy, and with the serum concentration of anti-endomysial antibodies. Recently, a longitudinal study revealed diminished levels of PRL after 6 months following a gluten-free diet. The evidence of decreasing PRL simultaneously with the decline of anti-transglutaminase antibodies suggests a direct connection with a gluten-free diet and hormone levels (112).

#### PRL and Autoimmune Thyroid Disease

Autoimmune thyroid diseases comprise mainly two disorders, Grave’s disease and Hashimoto thyroiditis (113). Hyperprolactinemia was found in 20% of patients with autoimmune thyroid disease and had double the frequency among hypothyroidism patients. Around 90% of Hashimoto’s thyroiditis patients presented significantly higher PRL levels in association with decreased cortisol titers (114). The role of dopamine agonists in the treatment of autoimmune thyroid disease is yet to be determined.

#### PRL and Peripartum Cardiomyopathy

Peripartum cardiomyopathy is a congestive heart failure occurring in the last month of pregnancy or 5 months after delivery, in the absence of preexisting heart disease (115). The etiology of this disease remains unclear, although plausible causes have been proposed, such as nutritional deficiency, viral infections, stress-activated cytokines, pathological response to hemodynamic stress, inflammation, and autoimmune reactions (116). Evidence supports an active role of PRL in the pathophysiology of this disease. Increased oxidative stress leads to subsequent 16-kDa PRL production, impairing the cardiac vasculature and its metabolism, culminating in systolic heart failure (117, 118). Interestingly, the presence of autoantibodies against sarcomeric myosin and troponin I were detected among women with peripartum myocardiopathy, suggesting the presence of an underlying autoimmune disorder. In addition, these antibodies were associated with the severity of left ventricle dysfunction and lower rate of full cardiac recovery on follow-up (119). Interestingly, patients demonstrated an abnormal cytokine profile (increased levels of TNF, IL-6 and soluble Fas receptors), decreased levels of CD4+ CD25lo Tregs, a heightened level of fetal microchimerism, and a significant reduction in the plasma levels of progesterone, estradiol, and relaxin, contributing to abnormal immune responses and inflammatory processes (120, 121). Recently, dopamine agonists have shown promising results in the treatment of this disease, dramatically improving outcomes (58, 122–124). The 2010 European position statement does not encourage breastfeeding based on concerns regarding the perpetuation of PRL pathways (125).

## Conclusion

The dimorphism between genders in autoimmune diseases is believed to rely on sex hormones. PRL exerts a great influence in immune system modulation, mainly inhibiting the negative selection of autoreactive B lymphocytes and has been associated with the pathogenesis of several autoimmune disorders. During pregnancy and the lactation period, assorted autoimmune patients experience disease relapse, suggesting an active influence of PRL. Immunological studies of pregnant and postpartum women with autoimmune diseases offer a biologically rich opportunity to improve our understanding of the hormonal impact on disease relapse pathophysiology. Although the interest on the relationship between PRL, immune modulation, and autoimmune diseases has emerged in the past few years, more studies are required to further delineate the influence of PRL in autoimmune disease. Eventually, gut microbiome, immune cells transcriptome, and proteome might be the answers to those questions being unsolved to date.

### Highlights

- Susceptibility genes, epigenetic modifications, microbiome, and sex hormones are believed to be a mainstay of the gender asymmetry in autoimmune diseases.
- PRL influences the negative selection of autoreactive B cells, promoting their proliferation, survival, and antibody production.
- Hyperprolactinemia has been associated with several autoimmune diseases and is believed to play a crucial role in their pathogenesis.
- A significant association between PRL and disease flairs was found in systemic lupus erythematosus and rheumatoid arthritis.
- Dopamine agonists have been used in the treatment of many autoimmune diseases with great benefits.

## Author Contributions

VB, ZG, and YS contributed equally to the construction of this review.

## Conflict of Interest Statement

The authors declare that the research was conducted in the absence of any commercial or financial relationships that could be construed as a potential conflict of interest.

## Abbreviations

IFN, interferon; Ig, immunoglobulin; IL, interleukin; MAPK, mitogen-activated protein kinase; MHC, major histocompatibility complex; PRL, prolactin; PRLR, prolactin receptor; Th, T helper cells; TNF, Tumor necrosis factor; Treg, T regulatory cells; STAT1, Signal transducer and activator of transcription 1.
